# Address Delivered before the American Society of Dental Surgeons, at the Opening of Its Fourth Annual Meeting

**Published:** 1843-09-16

**Authors:** 


### BIBLIOGRAPHICAL NOTICES.

*Address delivered before the American Society of Dental Surgeons, at the opening of its Fourth Annual Meeting; Baltimore, July 18th,* 1843. By Chapin A. Harris, A. M., M. D., Doctor of Dental Surgery; Professor of Practical Dentistry in the Baltimore College of Dental Surgery; Member of the Medico-Chirurgical Faculty of Maryland, etc. etc. Baltimore: 1843. 8vo. pp. 22.

Our readers may readily conceive the pleasure we experience, not only in reading, but in noticing a production like this. At the present moment, when the "dog star rages," in "far and fierce delight," undisturbed by any visiter from the realms of Eolus to divide his sway, we feel but too often oppressed by the dull monotony of the beaten track of our severer duties, and as often are tempted to prove recreant and seek for repose and variety in the more fascinating paths of the literature of our science. Dr. Harris's brochure arrived most seasonably, and in the same spirit which induced us to welcome its appearance, we will attempt a feeble delineation to our readers of the gratification and instruction we have derived from its perusal.

Many of our readers may perhaps be unaware that once in every year, the "American Society of Dental Surgeons,"—"the only regularly organized and extensive association in the world(!) of the kind, meet. "The welcome return of the Anniversary meeting, the fourth of this Association," is the occasion of the present effusion; when,

"From widely separated sections of the country, from our various field of labour, usefulness and *profit*, with spared lives, and obligations a thousand-fold increased, of gratitude to the great Supreme Preserver, we meet to promote the great cause of humanity, by strengthening each others' hands and *accomplishing* ourselves more perfectly in an art and science so intimately connected with the functions of health in the human system, with the enjoyment of life and the perfection of beauty, as are the operations of Dental Surgery in ameliorating the sufferings and increasing the happiness of man."

"These annual re-unions of members of a time-honoured(!) profession," at a mighty shrine, "before which [they] are to renew the truthful vows of deep devotion to the great cause of human good," Dr. H. regards as both pleasant and grand, an opinion in which we should cordially agree, were we fortunate enough to be one of the train-band. A description of the results of this "annual action," embodies so much that is new and startling in physiological diction, that we are induced to transcribe it.

"In this annual action there is analogy to the arterial and venous circulation of the blood of the human system; thrown from the *central ganglion*, it rushes through the canals of the arteries, then into the veins, perambulating the extremities of the body, taking in the needful supply of oxygen from contact with the air in the lungs or near the surfaces—then returning to leave its richness in the *great treasury of life*, to deposit all its acquired energy at the *central seat of vitality*; so, at stated periods, the members of this Society come from their posts of observation to report progress at the centre, to throw their discoveries, improvements, the results of industry, research and experience into the common fund—the great joint stock of dental knowledge."

A hypercritical spirit might suggest some slight tendency to rhetorical exaggeration, but there is a business-like air about the style, which, to our mind, entirely redeems it from any such imputation.

"The great and sublime spirit of association" is urged in an honest and impassioned strain, which could not have failed to convince its auditory. We regret that our limits do not permit us to present, in extenso, the argument. We have room only to cull one or two of the choicest *morceaux*.

"The necessity of associated effort and influence becomes apparent, and the American Society of Dental Surgeons, kept up in peace and concert from generation to generation, shall become *like the Persian band of immortals*, a living member stepping in to sustain the station of each one who may fall in the exercise of his profession, and ripe with all the virtues of his calling, and an extensive usefulness among his fellow men."

There is much brilliant imagery in the few following lines.

"Already have the muses smiled from Helicon on dental toil, and the goddess Hygeia, blushed with a more rosy beauty at the song of enchantment which celebrated the achievements of the art, and its connection both with health and loveliness; and other poets shall arise, like Darwin, Marmont and Brown, to crown the pale brow of 'star-eyed science' with bays of poesy!"

The sound, good sense, and practical philosophy of the annexed remarks will be generally acknowledged. A certain occasional homeliness of phraseology, judiciously tempers its rather aromatic tendency.

"One such friendly visit oftentimes puts out a flame of rising anger that might have illuminated a wide district of country, and in its lurid glare shown only the dark and diseased shadows of the human heart—the black vortices of shame, revenge, and every ungenerous, unlovely passion which the tempter of man ever planted in the deserts of the soul. A man never shows his weakest and darkest points of character, until he gets into a bitter and most implacable controversy with another; and then, when he is pouring out gall and wormwood upon the naked head of his antagonist, and, as he supposes, *using him up*, root and branch, before he is aware, he finds the whole public in possession of the fact, destructive for ever of his own fame and reputation, that he is a quarrelsome, and unfriendly, and revengeful man; and he finds out too late that his great controversy has been against his own character, and that he has, indeed, come off most irretrievably triumphant! A little patience, forbearance and investigation will set most matters at rest before the winds howl from the wilderness, and the turbid waves of passion begin to roll."

It is with heartfelt regret that we are obliged to part with our author, a regret which our readers must no doubt share with us. Before doing so we must indulge in one more quotation. This organization, whose destinies, we are told, "Time only can unravel," has in anticipation of future times, when, in those great revolutions of the globe which geology teaches us occur, aristocracy shall be in the ascendant in this hemisphere, adopted a coat of arms. This insignium, which would be a godsend to the College of Heralds—"is a polyandrian column, based upon a rock, surmounted by the lamp of science, throwing its rays above and around, encircled by the cognomen of the Association." Our author indulges the hope of seeing three such pillars arising from different empires of the globe.

The italics here, as elsewhere, are our own.

"Against three such columns, so planted on the rock of truth, and so illuminated by the 'star-eyed' radiations of science, the waves of error might dash in anger, but they would dash in vain. *In storm or calm, in 'gloom or glory,' those same changeless lights would shine, those same polyandrian columns stand unshaken, and those same adamantine rocks beat back the affled surges, still bearing unmoved those glory-tipped pillars, as light-houses of scientific discovery, and as monuments of associated intellectual energy, devoted to the best interests of mankind.*"

